# Comprehensive Characterization of Microbial Community in the Female Genital Tract of Reproductive-Aged Women in China

**DOI:** 10.3389/fcimb.2021.649067

**Published:** 2021-09-16

**Authors:** Ningxia Sun, Haixia Ding, Hongjing Yu, Yixuan Ji, Xiuyue Xifang, Wenjuan Pang, Xiang Wang, Qing Zhang, Wen Li

**Affiliations:** ^1^Center for Reproductive Medicine, Changzheng Hosptial, Second Military Medical University, Shanghai, China; ^2^Shanghai Key Laboratory of Embryo Original Diseases, Center for Reproductive Medicine & Fertility Preservation Program, International Peace Maternity and Child Health Hospital, School of Medicine, Shanghai Jiao Tong University, Shanghai, China; ^3^SPH Sine Pharmaceutical, Laboratories Co., Ltd., Shanghai Engineering Research Center of Innovative Probiotic Drugs, Shanghai, China

**Keywords:** female genital tract, microbiota, uterine cavity, infertile, *Lactobacillus*

## Abstract

The microbiota in the human body play critical roles in many physiological and pathological processes. However, the diversity and dynamics of the female genital tract (FGT) microbiota have not been fully unveiled. In this study, we characterized the microbiome variations in reproductive-aged Chinese women, and we revealed that the cervicovaginal microbiota were dominated by *Lactobacillus*. Overall, the composition of microbiota in the uterine cavity was more diverse than that in the vagina and cervix. A positive correlation between *Lactobacillus iners* and *Lactobacillus crispatus* was observed in both the vagina and the cervix, suggesting that these two species might have a symbiotic relationship in the cervicovaginal microbiota. Moreover, we, for the first time, stratified the reproductive-aged Chinese women into subgroups, based on their microbiome profiles. Furthermore, we identified the bacteria whose abundance changed in the uterine cavity of infertile patients when compared with healthy controls, such as *L. iners* and *L. crispatus*. Functionally, the metabolism-related pathways, neurotrophin signaling pathway, and adipocytokine signaling pathway were predominantly dysregulated in the uterine cavity of infertile patients. In conclusion, we characterized a comprehensive microbial landscape in FGT, as well as their functional roles in female infertility of the Chinese population.

## Introduction

The microbiota in the human body play a critical role in maintaining our daily wellbeing and are associated with the pathogenesis of various diseases (Young, 2017). These communities of microorganisms can be found in the skin, respiratory tract, alimentary tract, and other tissue sites, each with their own functional capabilities (Group NHW et al., 2009).

Among the spectrum of microbial communities, the female genital tract (FGT) microbiota, mainly dominated by *Lactobacillus* species, are considered to be one of the simplest yet most important microbial communities, as up to 9% of the human microbiota colonize the FGT, and the cervicovaginal microbiota have unique impacts on the reproductive health of women (Witkin and Linhares, 2017). For example, lactobacilli help regulate the pH of the vagina to inhibit the growth of other bacteria and to prevent undesirable microbial colonization and infection through their adhesion to the vaginal epithelial cells (Boris and Barbes, 2000). However, the composition, diversity, and dynamics of the microbiota in the uterine cavity of reproductive-aged women have not been fully unveiled, and as the uterine cavity is an essential part of the FGT, more efforts are needed to further illustrate the interaction between microbial communities in the vagina and uterus. Evidences have shown that FGT microbial communities are closely associated with gynecological diseases (White et al., 2011; Ma et al., 2012; Greenbaum et al., 2019). However, the impact of microbial communities in the uterine cavity on female fertility and the underlying mechanism are still unclear.

With 16S rRNA gene sequencing, a method that does not rely on microbial cultivation, it is possible to determine the composition of the microbiota in the FGT (Johnson et al., 2019). A previous study has investigated the microbial compositions in three sites of the vagina, including introitus, midpoint, and posterior fornix, and concluded that there was little variation in species across the three sampling sites, with *Lactobacillus* species being dominant in all sites (Human Microbiome Project C, 2012). Furthermore, a uterine microbiome study found that there was no difference in the microbial composition of infertile women who became pregnant and of those who did not undergo assisted reproductive technology (ART) (Franasiak et al., 2016). In addition, an analysis of the microbiota within the female reproductive tract revealed a microbiota continuum along the female reproductive tract, which is indicative of a non-sterile environment (Chen et al., 2017). In the present study, we measured the abundance of bacterial genera and species from the vagina, the endocervix, and the uterine cavity of reproductive-aged women; explored potentially competitive and symbiotic relationships within these microbial communities; and examined their biological functions, anticipating to uncover the association between microbial communities in reproductive-aged women and the female reproductive health.

## Materials and Methods

### Sample Collection

A total of 184 reproductive-aged women visiting the Physical Examination Center of Shanghai Changzheng Hospital were recruited for the study. Subjects with presence of an intrauterine device (IUD), vaginal inflammation, any acute inflammation, concern for cervical or endometrial neoplasia, and endocrine or autoimmune disorders were excluded. The subjects had no recorded recent use of hormones, antibiotics, and vaginal medications; no cervical treatment, endometrial biopsy, IUD removal, or hysteroscopy within a week; no douching within 5 days; and no sexual activity within 48 h. None of the subjects were pregnant, lactating, or menstruating at the time of sampling. Age, current resident area, menstrual history, and fertility history were collected from all the participants. As for nulliparous subjects, we asked whether they were planning for pregnancy. Written informed consent was obtained from all participants, and this study has been approved by the ethics committee of the Shanghai Changzheng Hospital.

Samples from the lower reproductive tract (vaginal and endocervix) were taken with a swab on the day of the visit with no prior perturbation. Endometrial fluid samples were obtained by transcervical aspiration with a double lumen embryo transfer catheter (Kitazato ET Catheter, Kitazato Corporation Tokyo office). Specifically, an outer cannula was placed at the internal cervical os. Subsequently, the inner sheath absorbed intrauterine lavage fluid and then retracted into the outer cannula. Finally, the inner sheath and the outer cannulas exited from the cervix and vagina together, thereby avoiding the contamination from bacteria in the cervix and vagina (Franasiak et al., 2016). The specimens were collected in sterile tubes, then flash-frozen with liquid nitrogen, stored at −80°C, and transported in dry ice to BGI-Shenzhen.

### DNA Extraction and 16S rRNA Amplicon Sequencing

Genomic DNA extraction was performed as previously described (Qin et al., 2012). DNA of high quality was used for PCR amplification, where the V1–V3 primers and the PCR master mix were used. It is well known that the V1–V3 regions of the 16S rRNA gene have a higher resolution for lower-rank taxa (genera and species); therefore, using primers targeting these regions allows for a more precise distance-based clustering of reads, which were then clustered into species-level amplicon sequence variants (ASVs) (Bukin et al., 2019). Finally, the PCR products were purified using the magnetic bead of Agencourt AMPure XP and dissolved in elution buffer. The range of the fragment in the library was tested by Agilent 2100 Bioanalyzer. The libraries passing quality control were sequenced by HiSeq 2000 platform. The primers for the V1–V3 regions were listed as follows: 8F-‘AGAGTTTGAT[YM]TGGCTCAG’, 518R-‘ATTACCGCGGCTGCTGG’. Y and M represent bases C/T and C/G, respectively.

### Quantitative Real-Time Polymerase Chain Reaction

Concentration of DNA from uterine cavity samples of 15 infertile patients and 15 healthy controls, and four reagents for DNA extraction, product purification, exonuclease, and DNA sequencing, were measured spectrophotometrically using a NanoDrop 2000 (Thermo Fisher, USA). The four reagents were used as negative controls. The real-time PCR assay was performed using primers to amplify the 16S rRNA genes and beta-actin. The primers were listed as follows (Ma et al., 2013): *Lactobacillus crispatus*: forward primer 5′-AGCGAGCGGAACTAACAGATTTAC-3′, reverse primer 5′-AGCTGATCATGCGATCTGCTT-3′; *Lactobacillus iners*: forward primer 5′-AGTCTGCCTTGAAGATCGG-3′, reverse primer 5′-CTTTTAAACAGTTGATAGGCATCATC-3′; beta-actin: forward primer 5′-AAAAGCCACCCCACTTCTCT-3′, reverse primer 5′-CTCAAGTTGGGGGACAAAAA-3′. The 20-μl PCR mixture contained 1 μl of DNA sample, 1 μl of each primer, 6 µl of ultra-pure water and 12 µl of 2*SYBR Green Mix. The Eppendorf realplex system (Eppendorf, USA) was used with the thermal cycling profile of 95°C for 5 min, and 40 cycles of 95°C for 30 s, 56°C for 30 s, and 72°C for 30 s. Each sample had three technical replicates. The abundance of the bacterium was calculated by dividing the average CT value of 16S rRNA gene by that of beta-actin.

### Cleaning the Raw Sequencing Data

The following steps were carried out to process the raw data: 1) discarding the reads of low base-quality: set 30 bp as the window length, and if the average quality of the window is lower than 17, truncate the end sequence of reads from the beginning of the window and remove the reads whose final read length is lower than 75% of the original read length; 2) discarding the reads contaminated by adapters: the default adapter sequence has an overlap of 15 bp with the read sequence, set it to 15 bp, and allow a mismatch of 3; 3) discarding reads with Ns; and 4) discarding reads with low complexity: the length of consecutive occurrences of a base in reads is ≥10. The resulting fastq format data were termed as clean data.

### Amplicon Sequence Variants and Taxonomy Analysis

The 16S rRNA clean data were processed by DADA2 package (Callahan et al., 2016) in R. DADA2 provides a sensitive and specific workflow in amplicon sequencing. The DADA2 pipeline proceeds as follows: 1) filter and trim clean data: discarding reads at the first instance of a quality score less than or equal to 2; 2) remove duplicated sequence entries in fastq files; 3) merge paired reads; 4) learn the error rates and infer the sample composition using the error rates; 5) construct a sequence table; 6) remove chimeras; and 7) assign taxonomy using naive Bayesian classifier method and the RDP_16sRNA reference databases.

### Normalizing the Relative Abundance of Bacteria

For each sample, we normalized the abundance of each bacterium at a genus or species level by dividing its raw count by the total number of read counts. The resulting proportions for each bacterium were used as the normalized abundance. Furthermore, the bacteria that accounted for less than 0.5% were combined and termed as “others”. The bacteria that accounted for over 0.5% in at least two samples were retained for further data analyses.

### Diversity Analysis

The alpha-diversity analysis was performed by R vegan package (Dixon, 2003). The Shannon index was used to evaluate the alpha-diversity of the microbiota. The Sorensen index was calculated by dividing the number of shared bacteria between the two samples by the total number of bacteria from those two samples.

### The Functional Prediction of Microbiota

Tax4fun (Asshauer et al., 2015) package in R was used to estimate Kyoto Encyclopedia of Genes and Genomes (KEGG) Ortholog (KO) scores for each sample, and the scores were further used to predict the relative activity of KEGG pathways.

### The Differential Abundance Analysis

The microbiota abundances between two groups were compared using Wilcoxon rank-sum test. The *p*-values were adjusted by the Benjamini and Hochberg method to avoid multiple testing (Benjamini and Hochberg, 1995). The Kruskal–Wallis test was applied to make multiple comparisons between groups.

### The Unsupervised Clustering Analysis

The unsupervised clustering analysis was conducted in R hclust. The Euclidean distance was used to measure the distance between samples based on the bacterial relative abundances. The dendrogram was determined by Ward clustering algorithm.

## Results

### The Microbiome Landscape of the Vagina, Cervix, and Uterine Cavity

**Figure 1:**
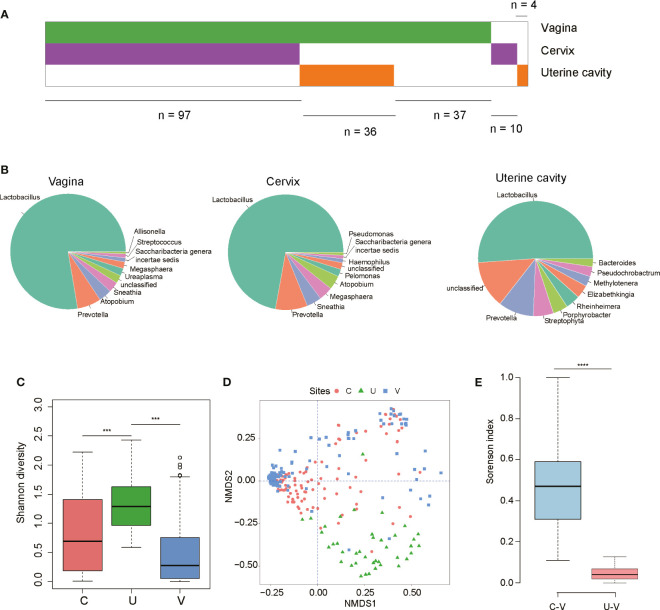
Microbiome landscape in the genital tract of reproductive-aged women. **(A)** An overview of the sample collections (97 paired vagina–cervix samples and 36 paired vagina–uterine cavity samples). **(B)** Pie chart for 10 genera with the highest abundance at each site according to the mean relative abundance. **(C)** The Shannon diversity among the three sites of female genital tract (FGT). **(D)** Comparison of community for non-metric dimensional scaling (NMDS) ordination of the Bray–Curtis distance between sampling sites (V, vagina; C, cervix; U, uterine). **(E)** Intraindividual similarity between vagina–cervix and vagina–uterine. ***P-value < 0.001, ****P-value < 0.0001.

To explore the microbiome landscape in the genital tract of reproductive-aged women, we recruited 184 reproductive-aged women and collected the swab samples from the vagina, the cervix, and the uterine cavity. Since some subjects refused to be sampled from the uterine cavity, ultimately, we only retained 170 vaginas, 107 cervix, and 40 uterine cavity samples for 16S rRNA sequencing with a stringent quality control (**Supplementary Data 1**). As shown in **Figure 1A**, 97 subjects had matched vagina and cervix samples, while 36 paired vagina and uterine cavity samples were collected from the same patients.

As the microbial samples in the uterine cavity had low biomass, we first excluded the bacteria potentially contaminated by “kitome” from a previous study (Salter et al., 2014). The microbiome profile analysis revealed that *Lactobacillus* and *Prevotella* were dominant in the three sites of reproductive-aged women (**Figure 1B** and **Supplementary Data 2**). Specifically, in 119 out of 170 vagina samples and 57 out of 111 cervix samples, *Lactobacillus* genus was observed to account for over 90% of the community. Moreover, the bacterial diversity within the microbiota was much higher in the uterine cavity than in both the vagina and cervix (**Figure 1C**). The non-metric multidimensional scaling (NMDS) analysis of the relative abundance of bacteria revealed that the vagina and cervix showed high similarity in the microbiota composition, while both their relative abundance was significantly different from that of the uterine cavity (**Figure 1D**). Furthermore, we measured the similarities of the microbial composition across the three sites of the reproductive tract by calculating the Sorenson indices between the paired samples from each individual. Consistently, higher similarities were observed in paired cervix–vagina samples than in the paired uterine cavity–vagina samples taken from the same recruits (**Figure 1E**, Wilcoxon rank-sum test, *p*-value < 0.05), suggesting that the microbial composition in the uterine cavity might be different from that in other sites of the female reproductive tract.

### Clustering and Characterization of the Reproductive-Aged Women by the Genital Tract Microbiomes

**Figure 2:**
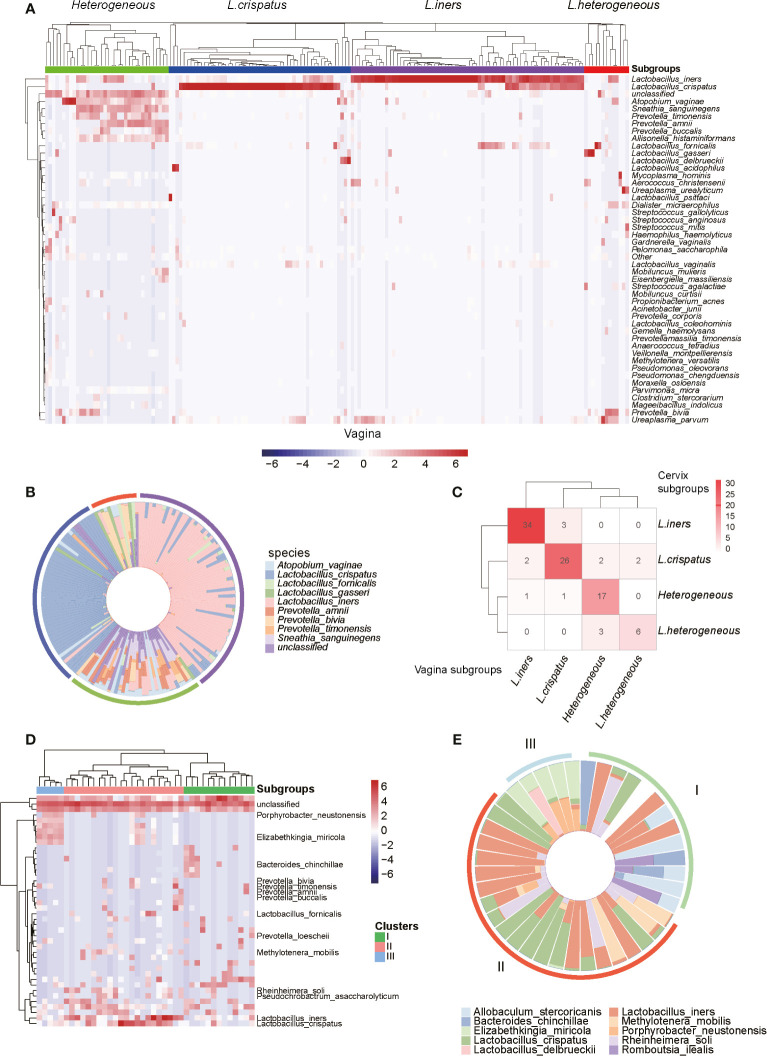
Microbiome profiles of the reproductive-aged women. **(A)** Heatmap of log10-transformed relative abundance of species found in the vaginal bacterial communities. **(B)** Species-level vaginal microbiome composition in each vagitype (10 species with the highest mean relative abundance were selected). **(C)** “Confusion matrix” was used to evaluate concordance between groupings of the vagina and cervix. **(D)** Heatmap of the uterine bacterial communities. **(E)** Species-level vaginal microbiome composition in each uterine microbiota communities type (10 species with the highest mean relative abundance were selected).

To further investigate the composition of microbiota in the genital tract of reproductive-aged women, we first conducted hierarchical clustering analyses for the vagina, cervix, and uterine cavity samples, which were based on the relative abundance of the bacteria at the species level (**Supplementary Data 2**). Overall, the vagina samples were classified into four groups denoted by I, II, III, and IV with 36, 47, 68, and 19 samples (**Figures 2A, B**), respectively. Group I was characterized by a large proportion of *Atopobium vaginae*, *Mobiluncus mulieris*, *Prevotella timonensis*, *Prevotella amnii*, and *Prevotella buccalis*. The vagina microbiota in groups II and III were dominated by *L. crispatus* and *L. iners*, which accounted for over 90% of the community in 44/47 and 41/68 samples of these two groups, respectively. Notably, group III and *L. iners* subgroup, as described in a previous study, exhibited consistent microbial composition (Ravel et al., 2011). In addition, group IV was dominated by several microbiomes such as *Lactobacillus fornicalis*, *Lactobacillus gasseri*, *Lactobacillus delbrueckii*, and *Lactobacillus acidophilus*. As a result, the four groups were termed as *Heterogeneous*, *L. crispatus*, *L. iners*, and *L.Heterogeneous*.

Similarly, the cervix (**Supplementary Figure 1**) and uterine cavity samples were also stratified into four and three subgroups using unsupervised clustering, respectively. Among the 97 subjects with paired vaginal and cervical samples, 34, 26, 17, and 6 subjects were classified as *L. iners*, *L. crispatus*, Heterogeneous, and L.Heterogeneous subgroups, respectively, based on both vaginal and cervical microbiota. Taken together, they accounted for 85.57% of total subjects and suggested that the two groupings were highly consistent (**Figure 2C**). However, the bacterial samples from the uterine cavity were clustered into three subgroups (**Figure 2D**), which greatly differed from both vaginal and cervical subgroupings. The first subgroup (subgroup I) in the uterine cavity was characterized by *L. iners* and *Allobaculum stercoricanis* (**Figure 2E**). Particularly, after investigating the clinical information of the samples, we found that all of those 10 infertile recruits were classified into this subgroup. The samples in subgroup II were colonized by two *Lactobacillus* species, *L. crispatus* and *L. iners* (**Figure 2E**). The samples in subgroup III were dominated by *Porphyrobacter neustonensis* and *Elizabethkingia miricola* (**Figure 2E**).

Furthermore, the alpha-diversity analysis of vaginal and cervical microbiomes revealed that the *L.Heterogeneous* and *Heterogeneous* groups showed higher diversity than the other two subgroups (**Supplementary Figure 2**, Kruskal–Wallis test, *p*-value < 0.0001). Similarly, the highest diversity was observed in subgroup I in the uterine cavity, followed by subgroups II and III (**Supplementary Figure 2**, Kruskal–Wallis test, *p*-value < 0.0001). From these results, we have disclosed that the microbiomes in the genital tract of reproductive-aged women varied greatly among individuals and body parts.

In addition, we also investigated the correlation between groupings and clinical characteristics. Specifically, we found that samples of *L. iners* had a slightly higher body mass index (BMI) than those of Heterogeneous subgroup (**Supplementary Figure 3**, *t*-test, *p*-value = 0.09).

### Site-Specific Microbiota in the Female Genital Tract

**Figure 3:**
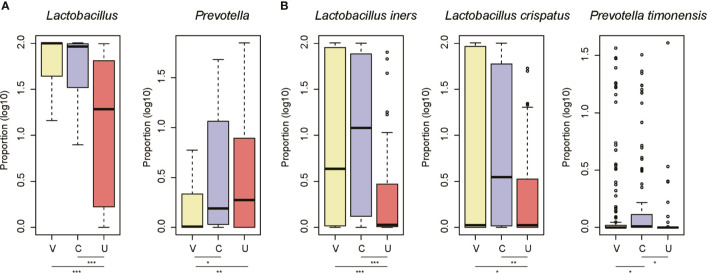
The site-specific genus and species in the three sites of female genital tract. The signature genus for the vagina/cervix **(A)** and uterine cavity **(B)**. The three sites are abbreviated as V, C, and U. The points on the top of the boxes represent the outliers. *P-value < 0.05, **P-value < 0.01, ***P-value < 0.001.

As the microbiota in the three sites of the FGT differed from one another, we then compared the microbiota between the three sites at both genus and species levels. Specifically, the proportions of *Lactobacillus* were higher in the vagina and cervix than the uterine cavity (**Figure 3A**, Wilcoxon rank-sum test, *p*-value < 0.05). In contrast, samples from the cervix and the uterine cavity had a higher proportion of *Prevotella* than those from the vagina (**Figure 3A**, Wilcoxon rank-sum test, *p*-value < 0.05). Further analyses of the species of the two genera revealed that *L. iners* and *L. crispatus* were more abundant in the vagina and cervix than the uterine cavity, while the cervix had a higher proportion of *P. timonensis* than the vagina and the uterine cavity (**Figure 3B**, Wilcoxon rank-sum test, *p*-value < 0.05). These results further indicated that the uterine cavity had a microbiome profile distinct from that of the vagina and cervix.

### Identification of the Microbiota With Changed Abundance in Infertile Women

**Figure 4:**
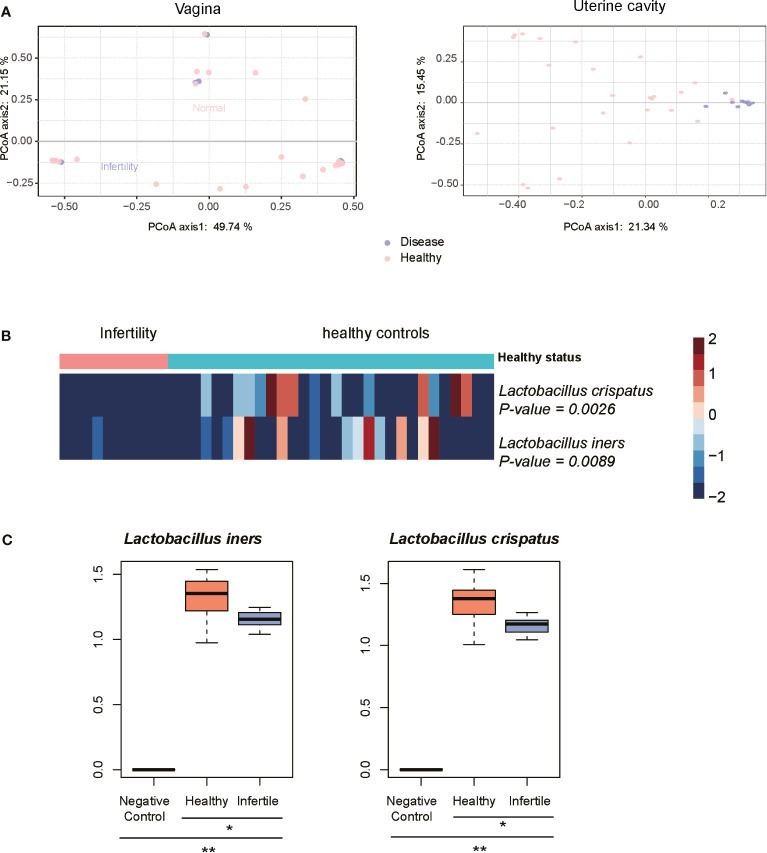
Characteristics of microbiota in infertility of reproductive-aged women. **(A)** Principal coordinates analysis (PCoA) on the Bray–Curtis distance at the species level for all taxonomic profiles at the vagina and uterine. **(B)** Heatmap of relative abundance of species scaled from −2 to 2 in the infertility and healthy controls communities. **(C)** The difference of abundances of two *Lactobacillus* species between the infertile patients, healthy controls, and negative controls by qPCR method. *P-value < 0.05, **P-value < 0.01.

As the 10 infertile patients were assigned to the same subgroup based on the composition of the uterine cavity microbiota, we then attempted to explore the microbiomes changed in these samples and their association with female infertility. As shown in **Figure 4A**, the infertile patients could be clearly differentiated from the healthy controls by their uterine cavity microbiota, but this difference was not observed when comparing their vaginal microbiota data using the principal coordinates analysis (PCoA). Similarly, the differences in the top two principal coordinates between infertile patients and healthy controls were also observed in the microbiota data of the uterine cavity (*p*-value < 0.05), but not in those of the vagina (the *p*-values of Wilcoxon rank-sum test for PC-1 and PC-2 were 0.16 and 0.98, respectively). These results indicated that it was not the microbiota in the vagina but the uterine cavity microbiota that have the potential to discriminate the infertile patients from healthy subjects and may be implicated in infertility.

Consistently, no certain bacterial species were observed to be significantly dominant or rare in the vagina of infertile patients (*p* < 0.05) when compared with healthy controls, further suggesting that the microbiota in the vagina of infertile patients and healthy reproductive-aged women showed no significant difference. On the contrary, *L. iners* and *L. crispatus* were significantly reduced in the uterine cavity of 10 infertile patients (**Figure 4B**, Wilcoxon rank-sum test, *p*-value < 0.05), suggesting that these bacterial species might be associated with female infertility. To verify the differential abundance of the two bacterial species between infertile patients and healthy controls, we quantified the abundance of these species in the uterine cavity of 15 infertile patients and 15 healthy controls using qPCR. Consistently, the abundances of two *Lactobacillus* species, *L. iners* and *L. crispatus*, were significantly decreased in the infertile patients as compared with the healthy controls (**Figure 4C**, *p*-value < 0.05). These results indicated that reduced abundance of *L. iners* and *L. crispatus* in the uterine cavity might be associated with female infertility.

### Functional Inferences of the Female Infertility by the Microbiota in the Uterine Cavity

**Figure 5:**
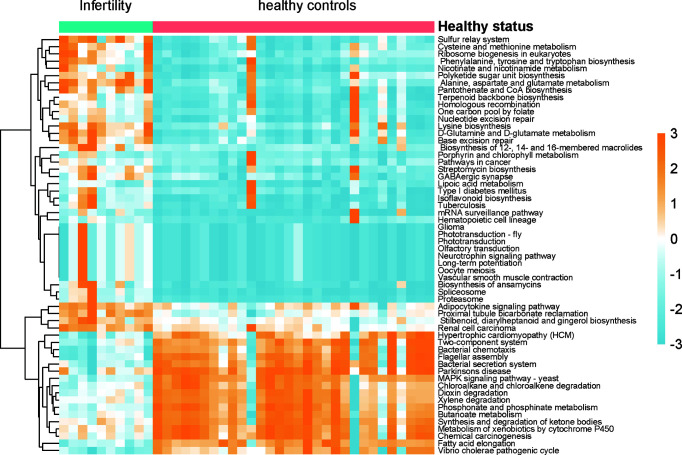
The differences of biological function between infertility and healthy controls of reproductive-aged women. The pathways were predicted by the relative abundances of microbiota, Wilcoxon test was used the compare the differences, and the resulting *p*-values were adjusted with false discovery rate (FDR) method.

As the microbiota in the uterine cavity were significantly altered in infertile patients, we aimed to explore the potential biological functions associated with this disease. The relative abundances of KO groups were estimated based on the microbiota in the uterine cavity by *tax4fun* method (**Supplementary Data 3**). Specifically, KOs in amino acid metabolism, metabolism of cofactors and vitamins, and biosynthesis of other secondary metabolites were observed to be more active in infertile patients (**Figure 5**). However, xenobiotics biodegradation and metabolism were observed to be downregulated in the uterine cavity, suggesting that the capability of degrading xenobiotics like chloroalkene and xenobiotics metabolized by cytochrome P450 in infertile patients might be decreased. Notably, two signaling pathways, neurotrophin signaling pathway and adipocytokine signaling pathway, were significantly enriched by those bacterial species found in infertile patients. Both neurotrophin and adipocytokine were secreted proteins and involved in the regulation of several physiological and pathological processes, indicating their potential implications in the pathogenesis of infertility.

## Discussion

The microbiota in the human body play a critical role in maintaining our daily wellbeing and are associated with several physiological and pathological processes. As it is difficult to directly sample the upper reproductive tract, the diversity of the microbiota in the FGT has not been fully unveiled.

In this study, we conducted a strict sampling method to avoid the bacterial contamination between the vagina and the uterine cavity, aiming to characterize the microbiome variations of the female reproductive tract, especially of the uterine cavity, in reproductive-aged Chinese women. The microbiome profiling revealed that *Lactobacillus* accounted for the majority of the microbiota in the vagina and cervix of reproductive-aged women. In 119 of 170 vagina samples and 57 of 111 cervix samples, *Lactobacillus* was observed to comprise over 90% of detected species. It is well-recognized that *Lactobacillus* is a protective bacteria in the FGT, and its reduced proportion is associated with several gynecological and obstetric disorders like infertility (Moreno and Simon, 2018), preterm delivery (Elovitz et al., 2019; Fettweis et al., 2019), and even gynecological cancers (Wang et al., 2018; Nene et al., 2019).

The uterine cavity has been classically considered a sterile site (Simon, 2018). Based on the 16S rRNA gene sequencing analysis, we found that the uterine cavity was dominated by several microbiomes, such as *Lactobacillus*, *Prevotella*, *Streptophyta*, *Porphyrobacter*, and *Rheinheimera*. Compared with the vagina and cervix, the uterine cavity had a higher diversity and significantly distinct microbiota compositions. It has been reported that the uterine cavity microbial colonization originated from the vagina (Moreno and Franasiak, 2017), and cervical mucus with high concentrations of inflammatory cytokines, immunoglobulins, and peptides with antimicrobial properties could result in lower biomass and higher diversity in the uterine cavity (Franasiak and Scott, 2015). Accordingly, our data indicated that the alpha-diversity of the uterine cavity microbiota was significantly higher than that of the vagina and cervix (**Supplementary Figure 2**), suggesting that the high diversity might be associated with low biomass caused by the antimicrobial effect of inflammatory factors in the FGT. The comparison of the microbiota abundance between the three sites of the FGT revealed that two genera, *Lactobacillus* and *Prevotella*, as well as their species including *L. iners*, *L. crispatus*, and *P. timonensis*, were enriched in one of the three sites. A previous study reported that *Lactobacillus* was rare in the endometrium and that its presence might be contaminated by vaginal *Lactobacillus* (Winters et al., 2019). To avoid this contamination, we used embryo transfer catheter to take samples from the FGTs following a previous study (Franasiak et al., 2016). Therefore, we speculated that *Lactobacillus* genus was rare but dominant in the uterine cavity.

Based on the relative abundances of the microbiota, reproductive-aged women were stratified by the microbiota detected in three sites of the reproductive tract. To our knowledge, this is the first study to stratify reproductive-aged women by the microbiome profiles in Chinese population. In accordance with the high similarity of microbiota compositions between the vagina and the cervix, the groupings based on the microbiota between the vagina and the cervix were also highly concordant. The differential abundance analysis revealed that subgroup I in the vagina and cervix samples was characterized by a high proportion of strictly anaerobic bacteria like *A. vaginae*, *M. mulieris*, *P. timonensis*, *P. amnii*, and *P. buccalis*; and a high consistency was found between group I and group IV, which was defined by a previous study (Ravel et al., 2011). The remaining subgroups had a high proportion of *Lactobacillus* genus and were dominated by unique *Lactobacillus* species. Notably, group III was consistent with the *L. iners* group reported by a previous study (Ravel et al., 2011). Similarly, the uterine cavity subgroups were dominated by different microbiomes. Particularly, all 10 infertile patients were clustered into the same subgroup, suggesting that the uterine cavity of the infertile patients might have unique microbial composition. Furthermore, the comparison of the microbiota in the three sites between the infertile patients and healthy controls revealed that the microbiome of the uterine cavity had more obvious difference between the patients and healthy controls than that of the vagina or cervix. To explore the microbiota with changed abundance in the uterine cavity of infertile female, we compared the uterine cavity microbiota of the 10 infertile patients with those of healthy controls. Unfortunately, the potentially pathogenic bacteria of female infertility dominant in uterine cavity still could not be accurately identified in this study due to the low biomass in the uterine cavity. In contrast, the proportions of two probiotic bacteria, *L. iners* and *L. crispatus*, were significantly reduced in the uterine cavity of 10 infertile patients, which had been verified by qPCR method. Collectively, decreased proportions of *L. iners* and *L. crispatus* were associated with female infertility (Zhang et al., 2020).

Functionally, microbiota-related KO predictions for the uterine cavity revealed that metabolism-related pathways were predominantly dysregulated in infertile patients. Moreover, two signaling pathways, neurotrophin signaling pathway and adipocytokine signaling pathway, were significantly enriched by the microbiota in infertility patients. Notably, neurotrophins, brain-derived neurotrophic factor (BDNF), and nerve growth factor (NGF) in follicular fluid of women have been used for different infertility diagnoses (Sadeu et al., 2012).

However, the present study still has some limitations. First, the contaminations could not be excluded thoroughly due to lack of negative controls. Second, higher sequencing depth is necessary for the low-biomass samples. Overall, we characterized a comprehensive landscape of the microbiome in Chinese female reproductive tract, as well as explored the microbiota with changed abundance in infertile and biological functions potentially involved in female infertility. In summary, the present study provided microbiota reference for the healthy reproductive-aged women and had potential values for scientific and clinical applications in female infertility.

## Data Availability Statement

The data presented in the study are deposited in the The National Omics Data Encyclopedia (NODE) repository, accession number OEP002094.

## Ethics Statement

The studies involving human participants were reviewed and approved by the ethics committee of Shanghai Changzheng Hospital. The patients/participants provided their written informed consent to participate in this study.

## Author Contributions

WL and NS designed this study. NS, HD and HY conducted the experiments. NS, XX, WP, and XW conducted the data analysis. NS, HD, and QZ performed the data visualization. NS, YJ, and HY contributed to the writing of the paper and setting of figures. All authors contributed to the article and approved the submitted version.

## Funding

This work was supported by the National Key R&D Program of China (2018YFC1002802); the National Natural Science Foundation of China (81901482 and 81873821); and Military innovation project special project (18JS009).

## Conflict of Interest

Author HY was employed by company SPH Sine Pharmaceutical, Laboratories Co., Ltd.

The remaining authors declare that the research was conducted in the absence of any commercial or financial relationships that could be construed as a potential conflict of interest.

## Publisher’s Note

All claims expressed in this article are solely those of the authors and do not necessarily represent those of their affiliated organizations, or those of the publisher, the editors and the reviewers. Any product that may be evaluated in this article, or claim that may be made by its manufacturer, is not guaranteed or endorsed by the publisher.

## Supplementary Material

Supplementary Figure 1 The microbiome profiles of the cervix in reproductive-aged women. **(A)** The heatmap of log10-transformed relative abundance of species found in the cervical bacterial communities. **(B)** Species-level vaginal microbiome composition in cervix (ten species with the highest mean relative abundance were selected).

Supplementary Figure 2 The difference of alpha-diversity between the subgroups of vagina, cervix, and uterine.

Supplementary Figure 3 The association of the groupings with the clinical characteristics.
