# Supplementary figures and images for: Comprehensive Characterization of Microbial Community in the Female Genital Tract of Reproductive-Aged Women in China
Source: Front Cell Infect Microbiol. 2021 Sep 16;11:649067. doi: 10.3389/fcimb.2021.649067 (PMC8482844; doi:10.3389/fcimb.2021.649067)

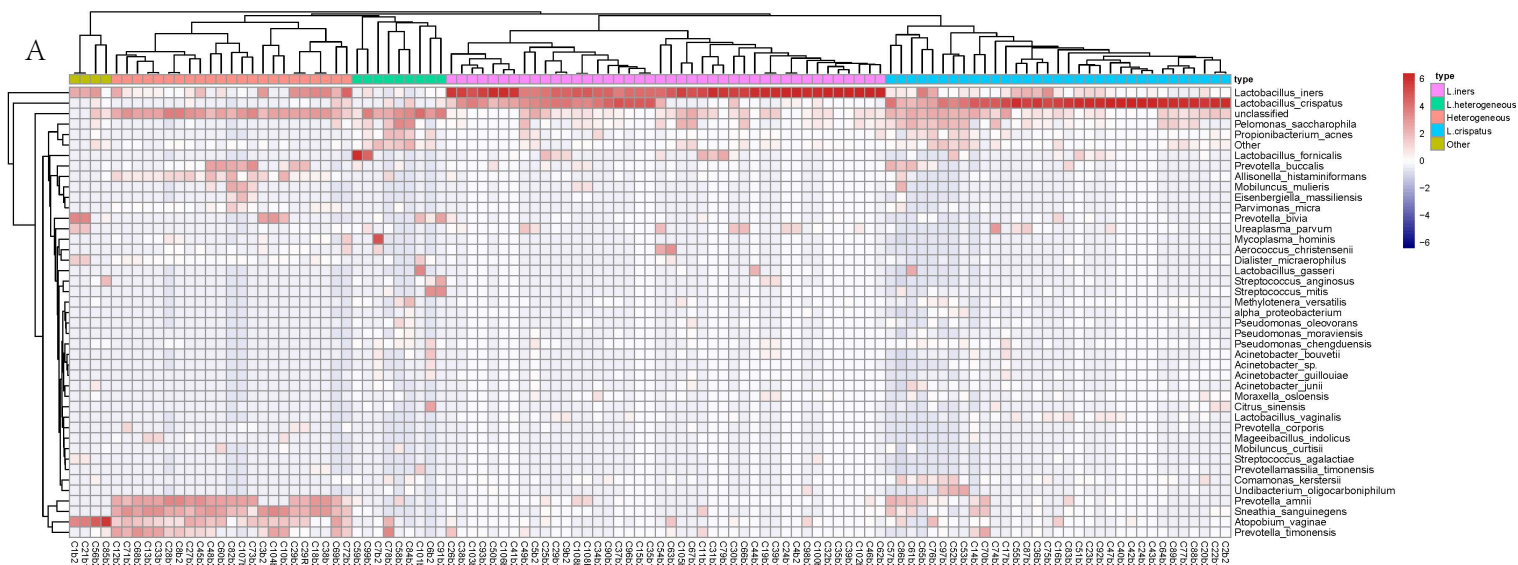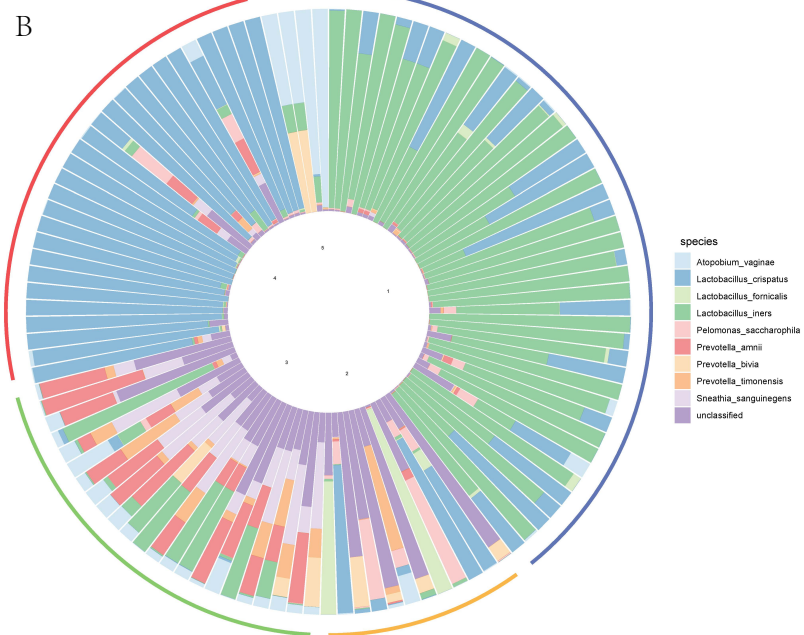

Supplement: Supplementary Figure 1 — The microbiome profiles of the cervix in reproductive-aged women. (A) The heatmap of log10-transformed relative abundance of species found in the cervical bacterial communities. (B) Species-level vaginal microbiome composition in cervix (ten species with the highest mean relative abundance were selected). [file Image_1.pdf]

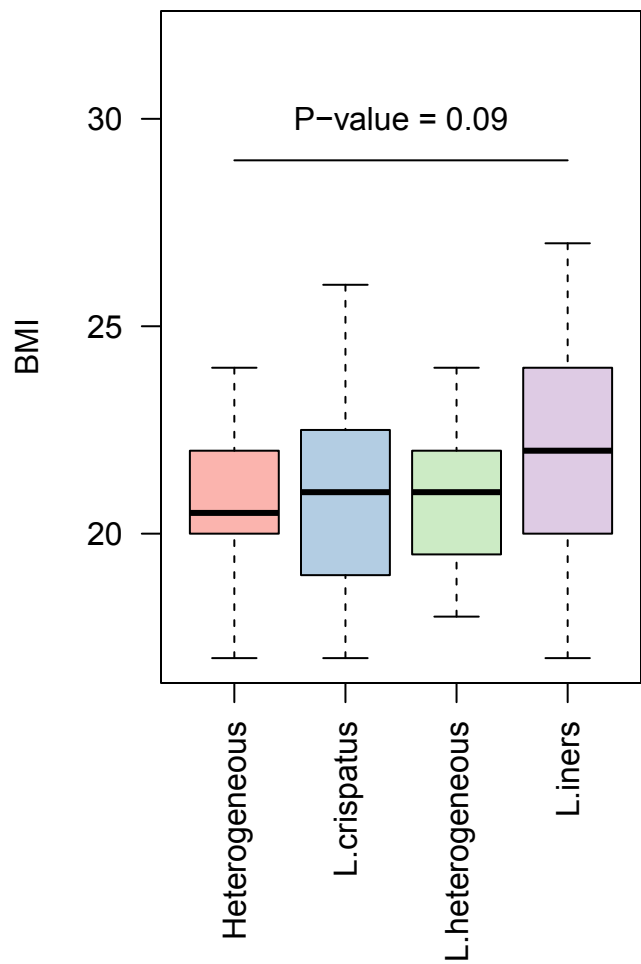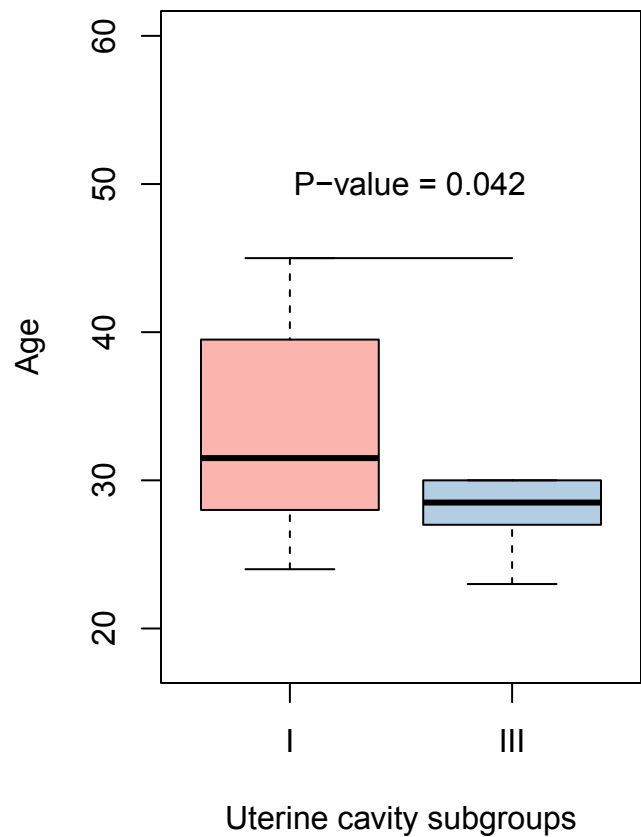

Supplement: Supplementary Figure 3 — The association of the groupings with the clinical characteristics. [file Image_3.pdf]
