# Supplementary material for: Comprehensive Characterization of Microbial Community in the Female Genital Tract of Reproductive-Aged Women in China
Source: Front Cell Infect Microbiol. 2021 Sep 16;11:649067. doi: 10.3389/fcimb.2021.649067 (PMC8482844; doi:10.3389/fcimb.2021.649067)
Supplement: Supplementary Figure 2 — The difference of alpha-diversity between the subgroups of vagina, cervix, and uterine. [file Image_2.pdf]

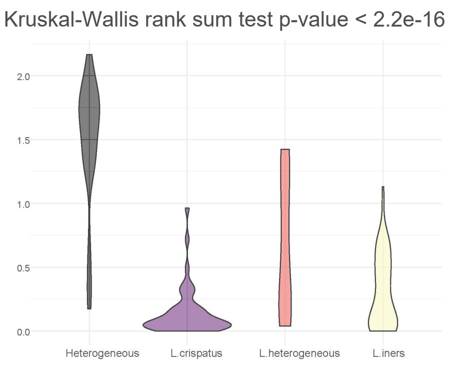


V Shannon Index

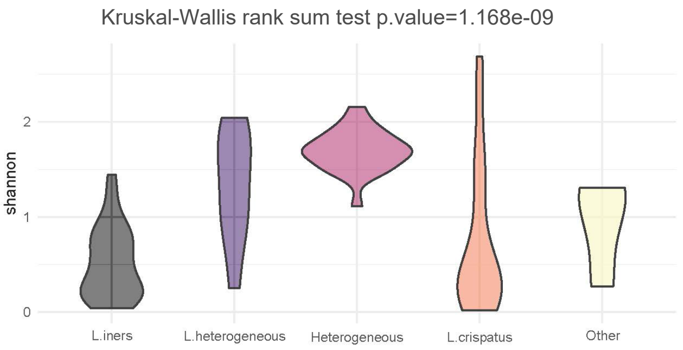


C Shannon Index

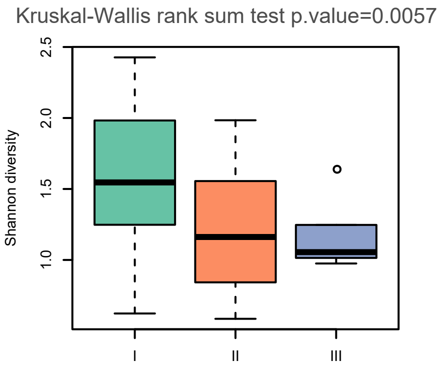


U Shannon Index
